# Supplementary material for: Changes in corneal astigmatism and near heterophoria after smartphone use while walking and sitting
Source: PLoS One. 2020 Dec 3;15(12):e0243072. doi: 10.1371/journal.pone.0243072 (PMC7714198; doi:10.1371/journal.pone.0243072)
Supplement: Attachment — Submitted filename: Response to reviwers comments.docx [file pone.0243072.s003.docx]

**Reviewer #1:**

Well done. You've done a great job revising this manuscript. I especially like the inclusion of Table 2.

Thank you very much for reviewing this manuscript and providing valuable comments.

**Reviewer #2:**

Thank you for the revision. Only one additional query has come up. When watching the movie on the smart phone, were they watching a movie in a language that required them to read the subtitles? In other words, were the subtitles an essential part of the experimental condition?

Thank you very much! It’s an excellent question. Sorry that we have missed it in the previous version. We played a Korean variety show called “Running Man”, a popular foreign TV program in Hong Kong, for 30 minutes with Chinese subtitle (subtitle’s size: ~ 3mm). None of the participants knew Korean, and reading the subtitle was necessary. This information has been added in the revised version (P7, L159-161).
